# Neoagarotetraose Alleviates Atherosclerosis via Modulating Cholesterol and Bile Acid Metabolism in ApoE^−/−^ Mice

**DOI:** 10.3390/nu16101502

**Published:** 2024-05-16

**Authors:** Junyi Li, Shaoqing Yang, Dan Liu, Qiaojuan Yan, Huiyuan Guo, Zhengqiang Jiang

**Affiliations:** 1 Key Laboratory of Food Bioengineering (China National Light Industry), College of Food Science and Nutritional Engineering, China Agricultural University, Beijing 100083, China; b20193060509@cau.edu.cn (J.L.); ysq@cau.edu.cn (S.Y.); b20213060519@cau.edu.cn (D.L.); 2 College of Engineering, China Agricultural University, Beijing 100083, China; yanqj@cau.edu.cn; 3 Department of Nutrition and Health, China Agricultural University, Beijing 100083, China; guohuiyuan@cau.edu.cn; 4 Food Laboratory of Zhongyuan, Luohe 462000, China

**Keywords:** atherosclerosis, neoagarotetraose, cholesterol synthesis, bile acid metabolism, gut microbiota

## Abstract

**Highlights:**

**What are the main findings?**

- Neoagarotetraose inhibited atherosclerosis and decreased the atherosclerotic le-sion area by 50.1%.
- Neoagarotetraose exerted regulatory effects on metabolic pathways related to both cholesterol and bile acid metabolism.

**What is the implication of the main finding?**

- The main finding highlighted the role of gut microbiota in neoagaro-tetraose-mitigating atherosclerosis in HFHCD-fed ApoE^−/−^ mice.
- The main finding demonstrated the potential application of NAT as a novel die-tary supplement to prevent AS.

**Abstract:**

Atherosclerosis is closely associated with metabolic disorders such as cholesterol accumulation, bile acid metabolism, and gut dysbiosis. Neoagarotetraose supplementation has been shown to inhibit obesity and alleviate type 2 diabetes, but its effects on modulating the development of atherosclerosis remain unexplored. Therefore, the present study was conducted to investigate the protective effects and potential mechanisms of neoagarotetraose on high-fat, high-cholesterol diet (HFHCD)-induced atherosclerosis in ApoE^−/−^ mice. The results showed that neoagarotetraose supplementation decreased the atherosclerotic lesion area by 50.1% and the aortic arch lesion size by 80.4% compared to the HFHCD group. Furthermore, neoagarotetraose supplementation led to a significant reduction in hepatic lipid content, particularly non-high-density lipoprotein cholesterol. It also resulted in a substantial increase in total bile acid content in both urine and fecal samples by 3.0-fold and 38.7%, respectively. Moreover, neoagarotetraose supplementation effectively downregulated the intestinal farnesoid X receptor by 35.8% and modulated the expressions of its associated genes in both the liver and intestine. In addition, correlation analysis revealed strong associations between gut microbiota composition and fecal bile acid levels. These findings highlight the role of gut microbiota in neoagarotetraose-mitigating atherosclerosis in HFHCD-fed ApoE^−/−^ mice. This study indicates the potential of neoagarotetraose as a functional dietary supplement for the prevention of atherosclerosis.

## 1. Introduction

Atherosclerosis (AS), the leading cause of cardiovascular disease, is characterized by thickening of the arterial intima, the formation of foam cells, the development of plaque on vessel walls, and the potential for stenosis or complete blockage of the lumen [1,2]. The total cholesterol level is elevated, which is the central risk factor for AS [3]. Clinical studies have consistently demonstrated that reducing cholesterol content effectively inhibits the progression of AS [4,5]. Statins are widely used for preventing AS by inhibiting cholesterol biosynthesis and subsequently lowering plasma cholesterol levels, but they are potentially associated with adverse effects such as hyperglycemia and myopathies [3,6]. Therefore, it is of increasing importance to find effective prevention and treatment methods to slow down and reverse the pathological process of AS. The conversion of cholesterol to bile acid plays a critical role in maintaining cholesterol homeostasis and preventing the accumulation of cholesterol, triglycerides, and toxic metabolites [7]. Bile acid has been associated with cardiovascular diseases in both animals and humans [8,9]. The synthesis of bile acid is regulated by fasting, refeeding, and nutrient status [10]. Hence, dietary interventions regulating bile acid metabolism can impede the progression of AS [11,12,13,14].

Recent animal and clinical studies have demonstrated that the gut microbiota is a key regulatory factor for various metabolic diseases [4,15,16]. As an important class of microbiota-derived metabolites, bile acid modulates the composition of the gut microbiota [17]. Moreover, gut microbiota regulate bile acid metabolism to alleviate AS [12,13,14]. Resveratrol at a dose of 40 mg/kg bw/d regulated bile acid metabolism in C57BL/6J and ApoE^−/−^ mice to attenuate trimethylamine-N-oxide-induced AS with increased abundances of *Lactobacillus* and *Bifidobacterium* [12] Naringin alleviated AS in ApoE^−/−^ mice by promoting the synthesis of bile acid through the gut microbiota–liver–cholesterol axis [14] Intake of dioscin regulated cholesterol metabolism by adjusting farnesoid X receptor (FXR)-mediated liver–gut crosstalk of bile acid and thus inhibited AS in ApoE^−/−^ mice [13].

Functional oligosaccharides, as one kind of prebiotic, exhibit various biological activities in the host by regulating the gut microbiota [18,19,20,21]. Oral gavage of naringin fucoidan and galactooligosaccharides at doses of 100 and 800 mg/kg bw/d ameliorated dyslipidemia in Sprague–Dawley rats by modulating gut microbiota and bile acid metabolism [18]. Poria cocos oligosaccharides reshaped the unbalanced gut microbiota to improve glucolipid metabolism disorder in mice fed a high-fat diet, accompanied by the change of individual bile acid in feces [21]. Mannose oligosaccharide (MOS) derived from *Saccharomyces cerevisiae* decreased the level of cholesterol in plasma, interfered with the intestinal flora to increase the cecal butyric acid content, and promoted bile acid excretion to inhibit the development of AS [19]. Neoagarotetraose (NAT), derived from agarose [22], has a variety of functional activities, such as anti-obesity and anti-aging, and was found to extend the lifespan of Caenorhabditis elegans [23,24,25,26,27]. So far, NAT has demonstrated its efficacy in reducing lipid levels and modulating cholesterol metabolism in various metabolic disorders, along with its impact on gut microbiota regulation [23,25,26,27]. NAT supplementation may confer a potential anti-atherosclerotic therapeutic strategy. However, the role and mechanism of NAT on AS, particularly bile acid metabolism, have not yet been studied.

Therefore, we investigated the potential impact of NAT intervention on the progression of AS in high-fat, high-cholesterol diet (HFHCD)-fed ApoE^−/−^ mice, encompassing assessments of cholesterol metabolism, bile acid metabolism, and gut microbiota. The results explain the molecular mechanisms underlying the protective effects of NAT against AS and offer valuable insights into the potential application of NAT as a functional food supplement.

## 2. Materials and Methods

### 2.1. NAT Preparation

NAT was prepared according to the previous method [22]. The purity of NAT was 93.2% (*w*/*w*), which was determined by high-performance liquid chromatography.

### 2.2. Animal Experiment

Male ApoE^−/−^ mice (7-week-old) on a C57BL/6J background and C57BL/6J male mice (7-week-old) were obtained from Beijing Vital River Laboratory Animal Technology Co., Ltd. (Beijing, China). The mice were housed in the controlled environment with 40–60% humidity, a 12 h/12 h light/dark cycle, and a constant temperature of 23 ± 2 °C. This study used two types of diets purchased from KeAo XieLi Feed Co., Ltd. (Beijing, China): standard diet (10.0% kcal from fat, AIN93M) and HFHCD (45% kcal from fat, containing 0.2% (*w*/*w*) cholesterol, TD10885). After acclimation for one week, the ApoE^−/−^ mice were randomly divided into 3 groups (n = 10 each): ApoE^−/−^ mice were fed the standard diet (ApoE^−/−^ group); ApoE^−/−^ mice were fed HFHCD (HFHCD group); ApoE^−/−^ mice were fed HFHCD plus NAT at a dose of 1200 mg/kg bw/d (HFHCD + NAT group). Additionally, another group containing 10 male C57BL/6J mice was fed the standard diet (normal group). Mice in the HFHCD + NAT group received NAT through oral gavage for 12 weeks, while mice in the other groups received water via oral gavage. The food intake, water intake, and body weight of mice were monitored and calculated on a weekly basis. At the end of the 12-week experiment, urine and feces were collected. All mice were fasted for 12 h before euthanasia. The harvested blood was centrifuged (3000 rpm, 4 °C for 10 min) to separate the serum. The aorta, liver, epididymal fat, subcutaneous fat, perinephric fat, intestinal tissues, and cecal contents were collected. The weights of the liver, epididymal fat, subcutaneous fat, and perinephric fat in mice were recorded for further analysis. The study was conducted according to the Chinese national guidelines on the care and use of laboratory animals and approved by the Animal Ethics Committee of China Agricultural University (protocol code: 20185001-3; approval date: 27 November 2018).

### 2.3. Quantitative Analysis of Atherosclerotic Plaque Lesions

After heart perfusion, whole aortas were collected and fixed in 4% paraformaldehyde. Hearts were embedded and cryostat-sectioned at a thickness of 5 μm until three leaflets of aortic roots were visible. Sections of aortic roots were stained with Oil Red O (0.5%, *w*/*v*) to visualize lipid-rich areas or with hematoxylin and eosin (H & E) for general tissue morphology. Aortas were carefully cleaned of surrounding adventitia, opened longitudinally, and rinsed with 60% (*v*/*v*) isopropanol. Subsequently, the aortas underwent Oil Red O (0.5%, *w*/*v*) staining in isopropanol. To analyze the lesion area, the stained aorta samples underwent image analysis utilizing ImageJ 1.8.0 (NIH, Bethesda, MD, USA).

### 2.4. Measurement of Biochemical Indicators

The supernatants of hepatic homogenates were collected by centrifugation (3000 rpm, 4 °C for 20 min) for subsequent experiments. Levels of total cholesterol (TC), triacylglycerols (TG), high-density lipoprotein cholesterol (HDL-C), low-density lipoprotein cholesterol (LDL-C), aspartate aminotransferase (AST), and alanine aminotransferase (ALT) in both the serum and supernatants were measured using diagnostic kits (Nanjing Jiancheng Bioengineering Institute, Nanjing, China).

### 2.5. Measurement of Total Bile Acid

Liver tissues and fecal samples were homogenized in PBS (10%, *w*/*v*) and then centrifuged at 3000 rpm for 20 min at 4 °C. The supernatants were collected, and the levels of total bile acid (TBA) in the supernatants, serum, and urine were measured using spectrophotometric assays according to the manufacturer’s instructions (Nanjing Jiancheng Bioengineering Institute, Nanjing, China).

### 2.6. Histopathological Analysis

Liver and epididymal fat tissues were fixed in 4% paraformaldehyde. After dehydration, tissues were paraffin-embedded, sliced, and stained with H&E. Images were captured using a Nikon Eclipse E100 microscope (Tokyo, Japan). The epididymal adipose areas were quantified using ImageJ (NIH, Bethesda, MD, USA).

### 2.7. RNA Isolation and Gene Expression Quantitation

Total RNA was extracted from liver and colon tissues using the Trizol reagent kit (Life Technologies, Carlsbad, CA, USA), followed by reverse transcription into cDNA using the PrimeScript™ RT reagent kit (Takara, Dalian, China). The quantitative real-time quantitative polymerase chain reaction (RT-qPCR) was performed using the TB Green Premix Ex Taq kit (Takara, Dalian, China). The relative quantification of gene expression was carried out using the 2^−ΔΔCT^ method, and *Gapdh* was used as the internal control for normalization. The RT-qPCR primer sequences of target genes are shown in Table S1.

### 2.8. Western Blot

Liver and colon tissues were lysed in RIPA buffer (Roche Diagnostics Ltd., Mannheim, Germany) for 30 min. The methods have been described previously [20]. Primary antibodies against ATP-binding cassette subfamily G member 8 (ABCG8), hepatocyte nuclear factor 4α (HNF-4α), cytochrome P450 8b1 (CYP8B1), scavenger receptor class B type I (SR-BI), small heterodimer partner-1 (SHP-1), liver receptor homolog-1 (LRH-1), apical sodium-dependent bile acid transporter (ASBT), or β-actin were purchased from Abcam Inc. (Cambridge, UK). Primary antibodies against ATP-binding cassette transporter G1 (ABCG1), FXR, ATP-binding cassette subfamily G member 5 (ABCG5), bile salt export pump (BSEP), or oxysterol 7α-hydroxylase (CYP7B1) were purchased from ProteinTech Group Inc. (Chicago, IL, USA). Primary antibodies against 3-hydroxy-3-methyl glutaryl coenzyme A reductase (HMGCR), liver X receptor (LXR), sterol-regulatory element binding protein 2 (SREBP2), cytochrome P450 27a1 (CYP27A1), or cholesterol 7α-hydroxylase (CYP7A1) were purchased from Zengneng BioScience (Chengdu, China). Primary antibodies against Niemann–Pick C1-like 1 (NPC1L1) were purchased from Santa Cruz Biotechnology Inc. (Dallas, TX, USA). The membranes were incubated overnight with primary antibodies (1:1000 dilution) at 4 °C, followed by incubation with secondary antibodies at room temperature for 1 h. The signal was visualized using the ChemiDoc XRS system (Bio-Rad, Hercules, CA, USA), and the densitometry analysis of each protein band was conducted using ImageJ software (National Institutes of Health, Bethesda, MD, USA).

### 2.9. Gut Microbiota Analysis

The DNA was extracted using the E.Z.N.A.soil DNA kit with the beads beating step from the cecal contents. The bacterial V3–V4 regions of 16S rRNA genes were amplified using the primer pairs (338F/806R). The amplicons were analyzed using paired-end sequencing on an Illumina MiSeq platform (Illumina, San Diego, CA, USA). All sequences were clustered into amplicon sequence variants (ASVs) according to 100% similarity using the Usearch database (version 7.1). The relative abundance of each bacterial taxon was analyzed by QIIME 2.

### 2.10. Statistical Analysis

All data are presented as the mean ± standard deviation (SD). Statistical analysis was conducted using GraphPad Prism 8.0 (La Jolla, CA, USA). The distribution was determined by the Kolmogorov–Smirnov normality test. Statistical differences were measured using a one-way ANOVA with Tukey’s multiple comparison test. Results marked with different letters are significantly different (*p* < 0.05).

## 3. Results

### 3.1. NAT Supplementation Suppressed Atherosclerosis Development in ApoE^−/−^ Mice

**Figure 1:**
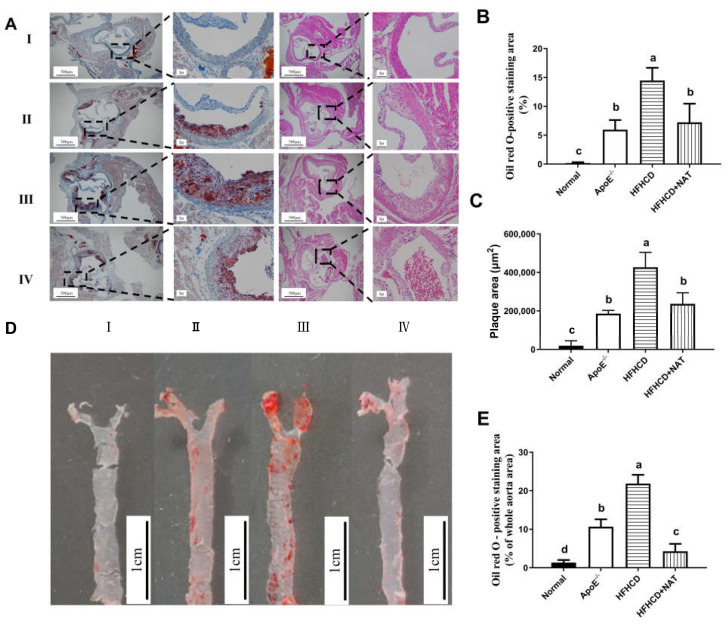
NAT ameliorated atherosclerotic lesions in HFHCD-fed ApoE^−/−^ mice. (**A**) Representative Oil Red O and H&E staining of aortic roots, I: normal group; II, ApoE^−/−^ group; III, HFHCD group; IV, HFHCD + NAT group. (**B**,**C**) Quantitative data of Oil Red O—positive staining area and plaque area in aortic roots. (**D**) Representative Oil Red O staining of aortas. (**E**) Quantitative data of Oil Red O—positive staining area in aortas. n = 5. Bars with different letters are significantly different (*p* < 0.05).

Oil Red O and H&E staining of aortic roots is shown in Figure 1A. Compared to the ApoE^−/−^ group, the mice fed with HFHCD displayed thickened aortic intima. Compared to the HFHCD group, NAT supplementation in the HFHCD + NAT group significantly reduced the aortic root lesion area (Figure 1B) and plaque area (Figure 1C) by 44.5% and 50.3%, respectively. Compared to the HFHCD group, Oil Red O staining of aortas demonstrated a reduction from 21.9 ± 3.1% to 4.3 ± 1.9% in the plaque area of the abdominal aorta in the HFHCD + NAT group (Figure 1D,E). These results suggested that NAT supplementation alleviated atherosclerotic lesions in HFHCD-fed ApoE^−/−^ mice.

### 3.2. NAT Supplementation Regulated Hepatic and Serum Lipid Levels in ApoE^−/−^ Mice

**Figure 2:**
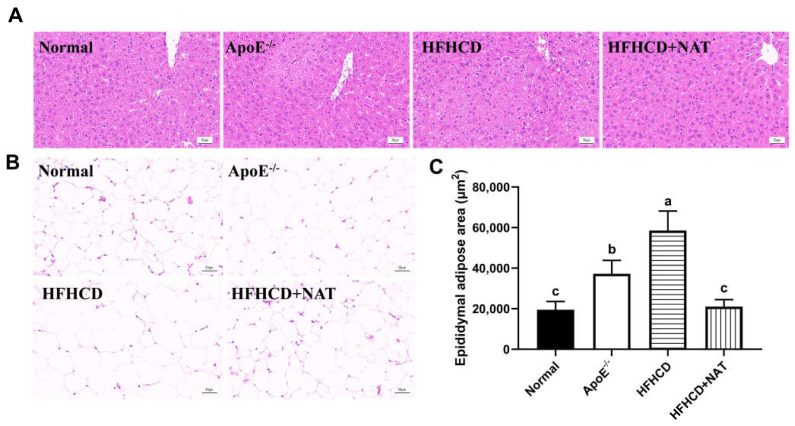
Effects of NAT supplementation on the liver and epididymal fat tissues. (**A**) H&E staining of liver tissues (magnification 200×). (**B**) H&E staining of epididymal fat tissues (magnification 200×). (**C**) Adipose area in epididymal fat tissues. n = 5. Bars with different letters are significantly different (*p* < 0.05).

Table 1 displays hepatic and serum lipids in different groups. The HFHCD group significantly increased serum and liver levels of TC and LDL-C, and there was a 96.4% increase in hepatic TG levels compared to the ApoE^−/−^ group. NAT supplementation led to a 20.0%, 35.9%, and 22.0% reduction in serum TC, TG, and LDL-C levels while significantly increasing serum and hepatic levels of HDL-C by 90.4% and 1.60-fold, respectively. Moreover, hepatic TC levels decreased by 41.2% after the NAT intervention. Compared to the HFHCD group, H&E staining of liver tissues in the HFHCD + NAT group revealed a significantly reduced degree of hepatic steatosis (Figure 2A) and a 64.1% decrease in the epididymal adipose area (Figure 2B,C). The body, liver, and fat weights of all mice are presented in Table S2. Compared to the HFHCD group, the HFHCD + NAT group displayed significant decreases of 8.1%, 10.1%, 25.6%, and 33.3% in body, liver, epididymal fat, and subcutaneous fat weights, respectively, but NAT supplementation did not reduce perinephric fat weight. Additionally, NAT supplementation significantly ameliorated HFHCD-induced increases in AST and ALT levels in serum. These results indicated that NAT regulated lipid levels in HFHCD-fed ApoE^−/−^ mice.

### 3.3. NAT Supplementation Ameliorated Cholesterol Metabolism in ApoE^−/−^ Mice

**Figure 3:**
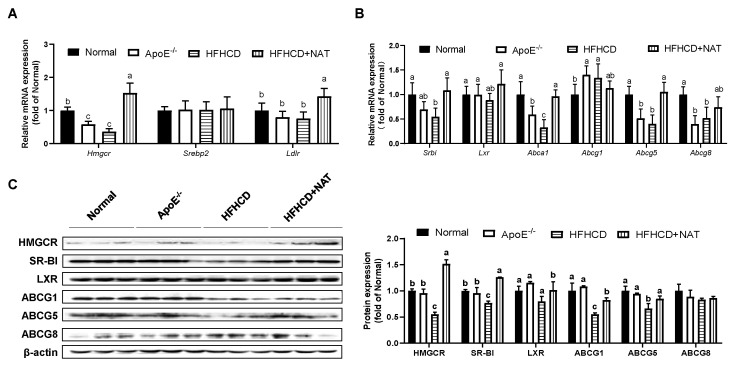
NAT mediated hepatic cholesterol metabolism in ApoE^−/−^ mice. (**A**) Hepatic mRNA expressions of genes involved in cholesterol uptake. (**B**) Hepatic mRNA expressions of genes involved in cholesterol synthesis and efflux. (**C**) Hepatic protein expressions of HMGCR, SR-BI, LXR, ABCG1, ABCG5, and ABCG8. n = 5. Bars with different letters are significantly different (*p* < 0.05).

**Figure 4:**
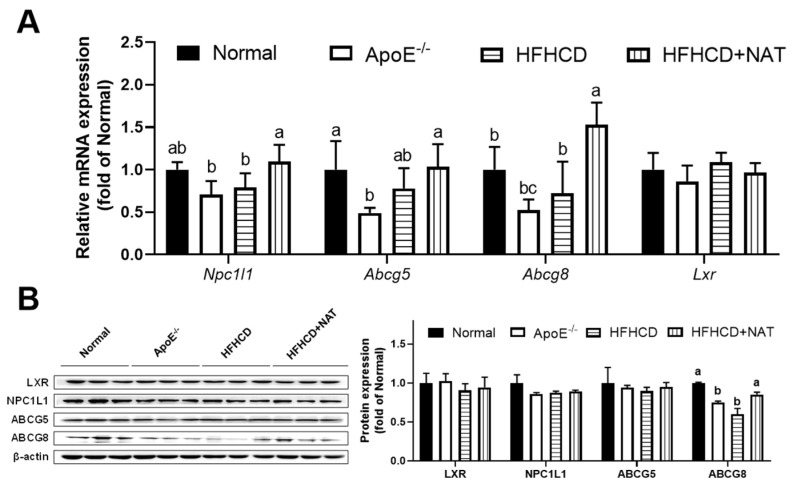
NAT mediated intestinal cholesterol metabolism in ApoE^−/−^ mice. (**A**) Intestinal mRNA expressions of genes involved in cholesterol uptake, synthesis, and efflux. (**B**) The intestinal protein expressions of LXR, NPC1L1, ABCG5, and ABCG8. n = 5. Bars with different letters are significantly different (*p* < 0.05).

The mRNA and protein expression levels of cholesterol metabolism-related genes in the liver and intestine were examined (Figure 3 and Figure 4). After the NAT intervention, hepatic mRNA expression levels of *Hmgcr* and *Ldlr* were upregulated by 3.21-fold and 89.2% (Figure 3A). NAT intervention increased mRNA expressions of cholesterol efflux-related genes, such as *Srbi* by 98.5%, *Abca1* by 1.9-fold, and *Abcg5* by 1.6-fold, but had no significant impact on hepatic mRNA expression levels of *Srebp2*, *Lxr*, *Abcg1*, and *Abcg8* (Figure 3A,B). Furthermore, hepatic protein expression levels of HMGCR, SR-BI, LXR, ABCG1, and ABCG5 in the HFHCD + NAT group were significantly upregulated by 173.8%, 63.6%, 27.4%, 49.7%, and 27.9%, respectively, compared to the HFHCD group (Figure 3C). However, hepatic protein expression levels of ABCG8 were not altered by NAT supplementation.

In the intestine, mRNA expression levels of *Npc1l1* and *Abcg8* were found to increase after NAT intervention. However, there were no significant alterations in the mRNA expression levels of *Abcg5* and *Lxr* following NAT supplementation (Figure 4A). Intestinal protein expression of ABCG8 increased by 41.9% after the NAT intervention. However, the protein expression levels of LXR, NPC1L1, and ABCG5 were not altered by NAT supplementation (Figure 4B). These results indicated that NAT supplementation improved hepatic and intestinal cholesterol metabolism in HFHCD-fed ApoE^−/−^ mice.

### 3.4. NAT Supplementation Ameliorated Bile Acid Metabolism in ApoE^−/−^ Mice

Compared to the ApoE^−/−^ group, the HFHCD group showed a 64.5% decrease in TBA content of feces, without significant changes in serum and liver TBA levels (Table 2). NAT supplementation in the HFHCD + NAT group increased TBA content in the serum and liver by 35.9% and 92.8%, respectively, compared to the HFHCD group. Furthermore, NAT supplementation significantly increased TBA content in urine from 1.4 to 5.7 mmol/L (Table 2).

**Figure 5:**
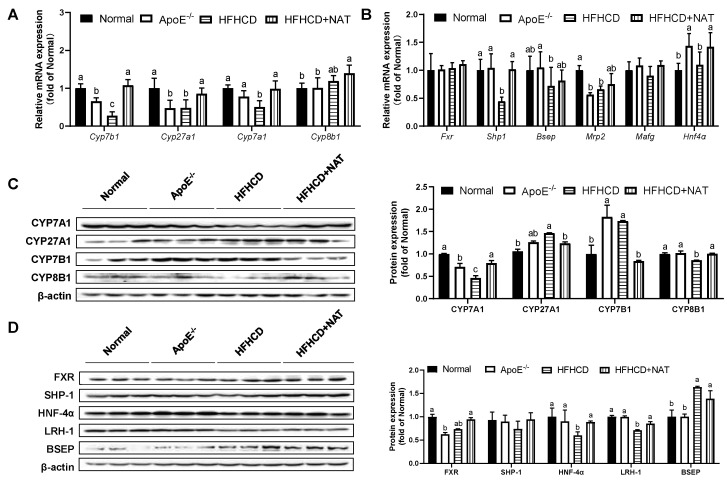
NAT mediated hepatic bile acid metabolism in ApoE^−/−^ mice. (**A**) Hepatic mRNA expressions of bile acid synthesis-related genes. (**B**) Hepatic mRNA expressions of FXR-related genes. (**C**) Hepatic protein expressions of CYP7A1, CYP27A1, CYP7B1, and CYP8B1. (**D**) Hepatic protein expressions of FXR, SHP-1, HNF-4α, LRH-1, and BSEP. n = 5. Bars with different letters are significantly different (*p* < 0.05).

**Figure 6:**
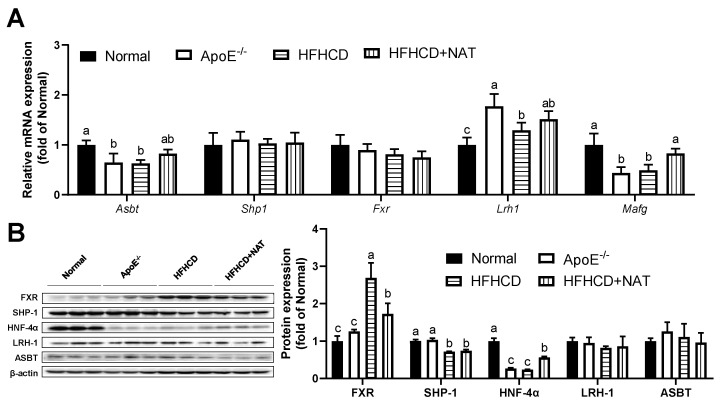
NAT mediated intestinal bile acid metabolism in ApoE^−/−^ mice. (**A**) The mRNA expressions of intestinal bile acid synthesis-related genes. (**B**) Intestinal protein expressions of FXR, SHP-1, HNF-4α, LRH-1, and ASBT. n = 5. Bars with different letters are significantly different (*p* < 0.05).

The impact of NAT on bile acid metabolism in the liver and intestine was examined (Figure 5 and Figure 6). Compared to the HFHCD group, NAT supplementation increased hepatic bile acid synthesis-related mRNA levels of *Cyp7b1*, *Cyp27a1*, and *Cyp7a1* by 2.9-fold, 78.9%, and 96.3%. However, the expression of *Cyp8b1* did not show a significant change (Figure 5A). NAT supplementation statistically increased hepatic mRNA expression levels of *Shp1* and *Hnf4α* by 1.29-fold and 29.5% compared to the HFHCD group (Figure 5B). NAT supplementation increased the hepatic protein expression levels of CYP7A1 and CYP8B1 by 71.5% and 16.4% (Figure 5C). In addition, NAT supplementation increased the hepatic protein expression levels of HNF-4α and LRH-1 by 47.2% and 20.5%, while the protein expression levels of FXR, SHP-1, and BSEP remained unchanged compared to the HFHCD group (Figure 5D).

In the intestine, NAT supplementation increased mRNA levels of *Mafg* by 68.3% while not changing mRNA levels of *Shp1*, *Fxr*, *Lrh1*, and *Asbt* compared to the HFHCD group (Figure 6A). Interestingly, a notable decrease in intestinal protein expression of FXR was observed after the NAT intervention (Figure 6B). NAT supplementation led to an increase in the protein expression level of HNF-4α by 90.6% in HFHCD-fed mice. However, the protein expression levels of SHP-1, LRH-1, and ASBT did not show significant changes between the HFHCD group and the HFHCD + NAT group (Figure 6B).

### 3.5. NAT Supplementation Regulated the Gut Microbiota in ApoE^−/−^ Mice

**Figure 7:**
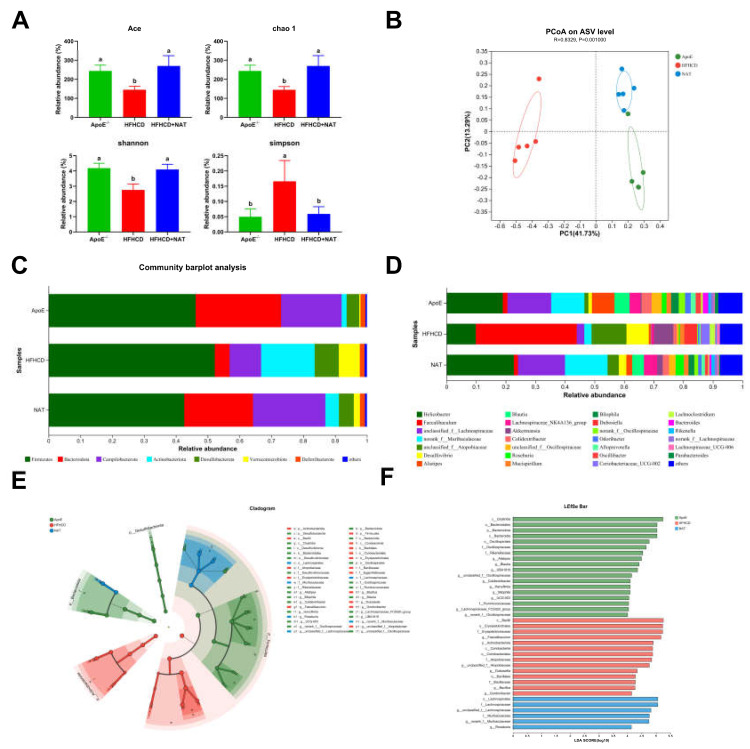
NAT supplementation altered the composition of the gut microbiota. (**A**) Analysis of α diversity metrics. (**B**) PCoA based on the abundance of ASVs. (**C**) Gut microbiota relative abundance at the phylum level. (**D**) Gut microbiota relative abundance at the genus level. (**E**) Gut microbiota diversity based on LEfSe analysis. (**F**) LDA scores. n = 5. Bars with different letters are significantly different (*p* < 0.05).

To investigate the effect of gut microbiota on NAT-induced inhibition of AS, gut microbiota analysis was performed in the ApoE^−/−^, HFHCD, and HFHCD + NAT groups. NAT supplementation increased the Ace, chao-1, and Shannon indices by 85.9%, 86.7%, and 48.8% while decreasing the Simpson index by 64.5% (Figure 7A). PCoA results showed significant differences in the composition and abundance of gut microbiota after the NAT intervention (Figure 7B). Bacterial populations were analyzed at both the phylum and genus levels (Figure 7C,D). At the phylum level, the relative abundance of Bacteroidota significantly decreased and Actinobacteriota increased in the HFHCD-fed mice compared to the standard diet-fed mice (Table S3). Compared to the HFHCD group, NAT supplementation resulted in an increased relative abundance of Bacteroidota and Campilobacterota and decreased Actinobacteriota in the HFHCD + NAT group (Table S3). At the genus level, relative abundances of *Faecalibaculum* and *Desulfovibrio* decreased and *norank_f_Muribaculaceae*, *g_unclassified_f_Lachnospiraceae*, and *Blautia* increased in the HFHCD + NAT group compared to the HFHCD group (Figure 7D and Table S4). LEfSe and LDA analysis identified *g_Faecalibaculum* as the keystone genus in the HFHCD group, while *g_unclassified_f_Lachnospiraceae* was the keystone genus in the HFHCD + NAT group (Figure 7E,F).

Spearman correlation analyses showed significant associations between the abundances of taxa and bile acid-related indices (Figures S1 and S2). These species were significantly correlated with at least one of the bile acid-related traits in the ApoE^−/−^ mice. Species that increased in the HFHCD group decreased with NAT supplementation. For example, *Faecalibaculum*, *Dubosiella*, and *unclassified_f__Atopobiaceae* were negatively associated with TBA content in feces (Figure S1). Additionally, species that were negatively related to intestinal protein expression of FXR were positively correlated to intestinal protein expression of HNF-4α (Figure S2). Thus, NAT supplementation regulated the gut microbiota and its relationship with bile acid metabolism, indicating its potential role in the inhibition of AS.

## 4. Discussion

Atherosclerosis, a primary contributor to cardiovascular disease, is characterized by the gradual buildup of arterial plaque within the walls of the arteries [1,3,6]. Regulation of bile acid metabolism is an effective approach to preventing hyperlipidemia and AS [9,11,12,13,28]. Functional oligosaccharides, such as galactooligosaccharides [29] and mannose oligosaccharides [19], improve hyperlipidemia and AS by regulating bile acid metabolism. However, the mechanism underlying how these oligosaccharides inhibit AS through bile acid metabolism remains unclear. NAT, originating from agarose [22], possesses regulatory capabilities over cholesterol and lipid metabolism [23]. The aim of this study was to explore the potential mechanism by which NAT affects AS.

Various studies have shown the regulatory impact of orally administered functional oligosaccharides on the development of AS [19,30]. Intervention with chitosan oligosaccharides for 12 weeks led to a decrease in plasma levels of LDL and VLDL in ApoE^−/−^ mice, leading to a reduction in AS progression [30]. In this study, NAT supplementation exhibited a noteworthy reduction in aortic lesion formation, accompanied by decreased circulating cholesterol levels and a decline in hepatic lipid accumulation (Figure 1 and Table 1). The regulatory effect of NAT might be associated with its ability to decrease cholesterol accumulation. As the primary hub of cholesterol homeostasis, the liver plays a vital role in cholesterol uptake, synthesis, and reverse cholesterol transport [5]. The HFHCD + NAT group exhibited significantly increased protein and gene expressions of HMGCR, a key mediator of cholesterol biosynthesis, compared to the HFHCD group. Moreover, the enhanced protein expression levels of LDLR and SR-BI, which are membrane proteins and crucial in lipoprotein metabolism [31], in response to NAT supplementation suggest an increased selective uptake of cholesterol from the bloodstream into hepatocytes (Figure 3). Additionally, cholesterol efflux, a crucial step in reverse cholesterol transport and a therapeutic target for AS, was effectively promoted by upregulating hepatic protein expression levels of ABCG8 in the presence of NAT, facilitating the transport of cholesterol to the gallbladder. Therefore, the findings indicate that NAT supplementation contributes to a reduction in lipid content in circulation and inhibits hepatic lipid accumulation by enhancing cholesterol uptake from the circulation and promoting cholesterol efflux to the intestinal lumen.

Cholesterol serves as a precursor for bile acid, which is essential for cholesterol metabolism [9]. Bile acid affects AS, with variations in bile acid content and excretion levels observed between AS and healthy individuals [32]. Dietary interventions that modulate bile acid metabolism have been explored as potential strategies to inhibit AS [13,19,33,34]. Dietary fiber intake facilitates the chelation of cholesterol and promotes its conversion into bile acid, leading to reduced hepatic cholesterol absorption and increased cholesterol efflux [35]. Accompanied by bile acid regulation, oral gavage of MOS derived from *S. cerevisiae* inhibited AS. But the mechanism by which MOS regulates bile acid has not been explored [19]. In this study, the HFHCD + NAT group demonstrated a significant rise in bile acid levels in the serum, liver, and urine compared to the HFHCD group (Table 2), indicating that NAT supplementation can regulate bile acid metabolism, thereby subsequently decreasing cholesterol accumulation in ApoE^−/−^ mice.

NAT exhibited distinct regulatory effects on key genes related to bile acid synthesis and transport in both the liver and intestine (Figure 5 and Figure 6). Moreover, NAT increased the hepatic mRNA expression levels of genes related to bile acid synthesis, including *Cyp7b1*, *Cyp27a1*, and *Cyp7a1*. NAT also upregulated the mRNA expression levels of *Shp-1* and *Hnf-4α*, which play crucial roles in regulating the bile acid transport pathway and accelerating hepatic bile acid efflux. In animal models, the activation of hepatic FXR and the inhibition of intestinal FXR have been shown to reduce AS [35,36]. Compared to the ApoE^−/−^ group, the HFHCD group exhibited a significant decrease in hepatic protein expression of FXR, while intestinal protein expression of FXR increased (Figure 5 and Figure 6). FXR serves as a main receptor for bile acid, and its modulation through bile acid supplementation has been associated with improvements in AS and hyperglycemia [11,36,37]. The intestinal FXR deficiency in ApoE^−/−^ mice led to smaller lesion areas in aortic roots and promoted hepatic cholesterol catabolism, indicating a potential therapeutic role for FXR in AS [36]. In the study, NAT supplementation resulted in decreased intestinal protein expression of FXR, along with upregulated HNF-4α expression, suggesting a reduction in bile acid reabsorption. Therefore, this regulation of FXR by NAT may be attributed to its impact on bile acid metabolism. Overall, these findings indicate that NAT exerts effects on AS partly through the modulation of bile acid metabolism, including increasing bile acid content and regulating key genes involved in bile acid synthesis and transport. The distinct roles of FXR signaling in the liver and intestine in response to NAT further highlight the complexity of bile acid-mediated mechanisms in AS development and the potential of NAT as a promising therapeutic agent in AS treatment.

As a novel prebiotic, NAT has been shown to effectively regulate gut microbiota [23,25,26,27]. NAT supplementation induced significant changes in the composition of the gut microbiota, leading to increased diversity (Figure 7). At the phylum level, there was a notable increase in the prevalence of Bacteroidota in the HFHCD + NAT group relative to the HFHCD group. These findings are consistent with a previous study on FXR^−/−^ mice fed a high-fat diet, which reported a lower relative abundance of Bacteroidota compared to wild-type mice on the same diet [38]. It is well established that microbial metabolism impacts the host’s bile acid profile, which in turn influences interactions with bile acid receptors such as FXR, thereby affecting overall health and disease development [39]. The relationship between bile acid metabolism and gut microbiota plays a significant role in AS development [7,10,12,19,28]. Metformin improves metabolic dysfunction through the *B. fragilis*–GUDCA–intestinal FXR axis, and the expression of FXR has been related to the gut microbiota [37]. To delve deeper into the impact of NAT on bile acid and gut microbiota, the relationships between NAT, bile acid, and gut microbiota were analyzed. Results demonstrated that all species were significantly correlated with at least one of the bile acid-related traits in the ApoE^−/−^ mice. *Alistipes*, a bacterial genus enriched in the gut microbiota of centenarians, has been shown to produce unique secondary bile acids [40]. *Alistipes* was found to be positively associated with bile acid content in feces (Figure S2) and showed enrichment in the HFHCD + NAT group compared to the HFHCD group. Additionally, NAT supplementation led to a significant increase in the relative abundance of *Blautia* at the genus level. *Blautia* is an anaerobic bacterium with probiotic characteristics known to produce deoxycholic acid and other secondary bile acids [41]. The above results implied that the regulation effect of NAT in bile acid was associated with the gut microbiota.

## 5. Conclusions

In conclusion, dietary supplementation with NAT (1200 mg/kg bw/d) effectively suppressed AS development in ApoE^−/−^ mice fed a HFHCD, resulting in a decrease in the aortic root lesion area by 44.5%. The anti-atherosclerotic effect of NAT is mediated through the interaction between gut microbiota and bile acid, especially the decreased intestinal FXR expression of 35.8%. These findings demonstrate the potential application of NAT as a novel dietary supplement to prevent AS and provide a novel approach for addressing this cardiovascular disease.

## Figures and Tables

**Table 1:** NAT altered hepatic and serum lipid profiles in ApoE^−/−^ mice.

	TC in Serum (mmol/L)	TG in Serum (mmol/L)	LDL-C in Serum (mmol/L)	HDL-C in Serum (mmol/L)	AST in Serum (U/L)	ALT in Serum (U/L)	TC in Liver (mmol/g)	TG in Liver (mmol/g)	LDL-C in Liver (mmol/g)	HDL-C in Liver (mmol/g)
Normal	2.4 ± 2.3 ^d^	1.7 ± 0.3 ^b^	0.4 ± 0.2 ^c^	0.9 ± 0.1 ^a^	13.8 ± 5.9 ^c^	48.9 ± 9.9 ^c^	4.4 ± 1.4 ^b^	2.6 ± 0.8 ^c^	0.9 ± 0.2 ^b^	1.1 ± 0.3 ^a^
ApoE^−/−^	10.4 ± 2.0 ^c^	2.3 ± 0.5 ^ab^	0.5 ± 0.2 ^c^	0.6 ± 0.1 ^b^	18.8 ± 4.6 ^bc^	59.9 ± 15.6 ^c^	5.0 ± 2.2 ^b^	5.6 ± 1.0 ^b^	1.0 ± 0.2 ^b^	0.3 ± 0.1 ^c^
HFHCD	19.0 ± 2.0 ^a^	2.9 ± 0.6 ^a^	1.3 ± 0.2 ^a^	0.5 ± 0.1 ^b^	36.1 ± 6.9 ^a^	11.0 ± 2.2 ^a^	8.8 ± 2.3 ^a^	11.0 ± 2.2 ^a^	2.1 ± 0.4 ^a^	0.3 ± 0.1 ^c^
HFHCD + NAT	15.2 ± 3.2 ^b^	1.9 ± 0.6 ^b^	1.0 ± 0.1 ^b^	1.0 ± 0.2 ^a^	22.9 ± 4.0 ^b^	91.2 ± 14.1 ^b^	5.2 ± 1.7 ^b^	9.3 ± 1.3 ^a^	1.7 ± 0.4 ^a^	0.7 ± 0.2 ^b^

n = 10. Bars with different letters are significantly different (*p* < 0.05).

**Table 2:** NAT ameliorated TBA contents in ApoE^−/−^ mice.

	TBA Content in Serum (mmol/L)	TBA Content in Liver (mmol/g)	TBA Content in Feces (mmol/g)	TBA Content in Urine (mmol/L)
Normal	5.57 ± 0.95 ^a^	1.22 ± 0.23 ^ab^	189.93 ± 23.96 ^a^	4.10 ± 0.75 ^b^
ApoE^−/−^	4.33 ± 1.04 ^b^	0.99 ± 0.12 ^b^	130.47 ± 35.91 ^b^	2.18 ± 0.83 ^c^
HFHCD	4.41 ± 0.75 ^b^	0.76 ± 0.31 ^b^	44.12 ± 8.29 ^c^	1.43 ± 0.25 ^c^
HFHCD + NAT	5.84 ± 0.81 ^a^	1.32 ± 0.15 ^a^	64.25 ± 11.02 ^c^	5.67 ± 0.98 ^a^

n = 10. Bars with different letters are significantly different (*p* < 0.05).

## Data Availability

The original contributions presented in the study are included in the article and Supplementary Materials, further inquiries can be directed to the corresponding author.

## Supplementary Materials

The following supporting information can be downloaded at: https://www.mdpi.com/article/10.3390/nu16101502/s1, Figure S1: Correlation analysis between identified bacterial species with bile acid indexes and mRNA expression levels of bile acid metabolism-related genes. The color gradient is shown from blue (low abundance) to red (high abundance), and “*” (*p* < 0.05) indicates significance; Figure S2: Correlation analysis between identified bacterial species with protein expression levels of bile acid metabolism-related genes. The color gradient is shown from blue (low abundance) to red (high abundance), and “*” (*p* < 0.05) indicates significance; Table S1: Primer sequences used for real-time quantitative polymerase chain reaction (RT-qPCR); Table S2: Effects of neoagarotetraose (NAT) on body, liver, and fat weights in HFHCD-fed ApoE^−/−^ mice; Table S3: Impact of neoagarotetraose (NAT) supplementation on the gut microbiota composition at phylum levels; Table S4: Impact of neoagarotetraose (NAT) supplementation on the gut microbiota composition at genus levels.

## Abbreviations

ABCG1, ATP-binding cassette transporter G1; ABCG5, ATP-binding cassette subfamily G member 5; ABCG8, ATP-binding cassette subfamily G member 8; ALT, alanine aminotransferase; AS, atherosclerosis; ASBT, apical sodium–dependent bile acid transporter; AST, aspartate aminotransferase; ASVs, amplicon sequence variants; BSEP, bile salt export pump; BSH, bacterial bile salt hydrolase; CYP7A1, cholesterol 7α-hydroxylase; CYP7B1, oxysterol 7α-hydroxylase; CYP8B1, cytochrome P450 8b1; CYP27A1, cytochrome P450 27a1; FGF-15, fibroblast growth factor 15; FXR, farnesoid X receptor; HDL-C, high-density lipoprotein cholesterol; HMGCR, 3-hydroxy-3-methyl glutaryl coenzyme A reductase; HNF-4α, hepatocyte nuclear factor 4α; H&E, hematoxylin and eosin; HFHCD, high-fat, high-cholesterol diet; LDA, linear discriminant analysis; LDL-C, low-density lipoprotein cholesterol; LDLR, low-density lipoprotein receptor; LRH-1, liver receptor homolog-1; LXR, liver X receptor; MOS, mannose oligosaccharides; MRP2, multidrug resistance-associated protein 2; NAT, neoagarotetraose; NPC1L1, Niemann–Pick C1-like 1; SHP-1, small heterodimer partner-1; SR-BI, scavenger receptor class B type I; SREBP2, sterol-regulatory element binding protein 2; SD, standard deviation; TBA, total bile acid; TC, total cholesterol; TG, triglyceride; RT-qPCR, real time-quantitative polymerase chain reaction.
