# Supplementary material for: Neoagarotetraose Alleviates Atherosclerosis via Modulating Cholesterol and Bile Acid Metabolism in ApoE−/− Mice
Source: Nutrients. 2024 May 16;16(10):1502. doi: 10.3390/nu16101502 (PMC11124046; doi:10.3390/nu16101502)
Supplement: Supplementary file 1 [file nutrients-16-01502-s001.zip › nutrients-3005480-supplementary.pdf]

Supporting information

Neoagarotetraoses Alleviates Atherosclerosis via Modulating Cholesterol and Bile Acids Metabolism in ApoE$^{-/-}$ Mice

Junyi Li [1], Shaoqing Yang [1], Dan Liu [1], Qiaojuan Yan [2], Huiyuan Guo [3], Zhengqiang Jiang [1,4,*]

[1] Key Laboratory of Food Bioengineering (China National Light Industry), College of Food Science and Nutritional Engineering, China Agricultural University, Beijing 100083, China; b20193060509@cau.edu.cn (J.L.); ysq@cau.edu.cn (S.Y.); b20213060519@cau.edu.cn (D.L.)

[2] College of Engineering, China Agricultural University, Beijing 100083, China; yanqj@cau.edu.cn

[3] Department of Nutrition and Health, China Agricultural University, Beijing 100083, China; guohuiyuan@cau.edu.cn

[4] Food Laboratory of Zhongyuan, Luohe 462000, Henan, China

* Corresponding author: Z.Q. Jiang

Tel.: +86-10-6273-7689; Fax: +86-10-8238-8508

E-mail: zhqjiang@cau.edu.cn

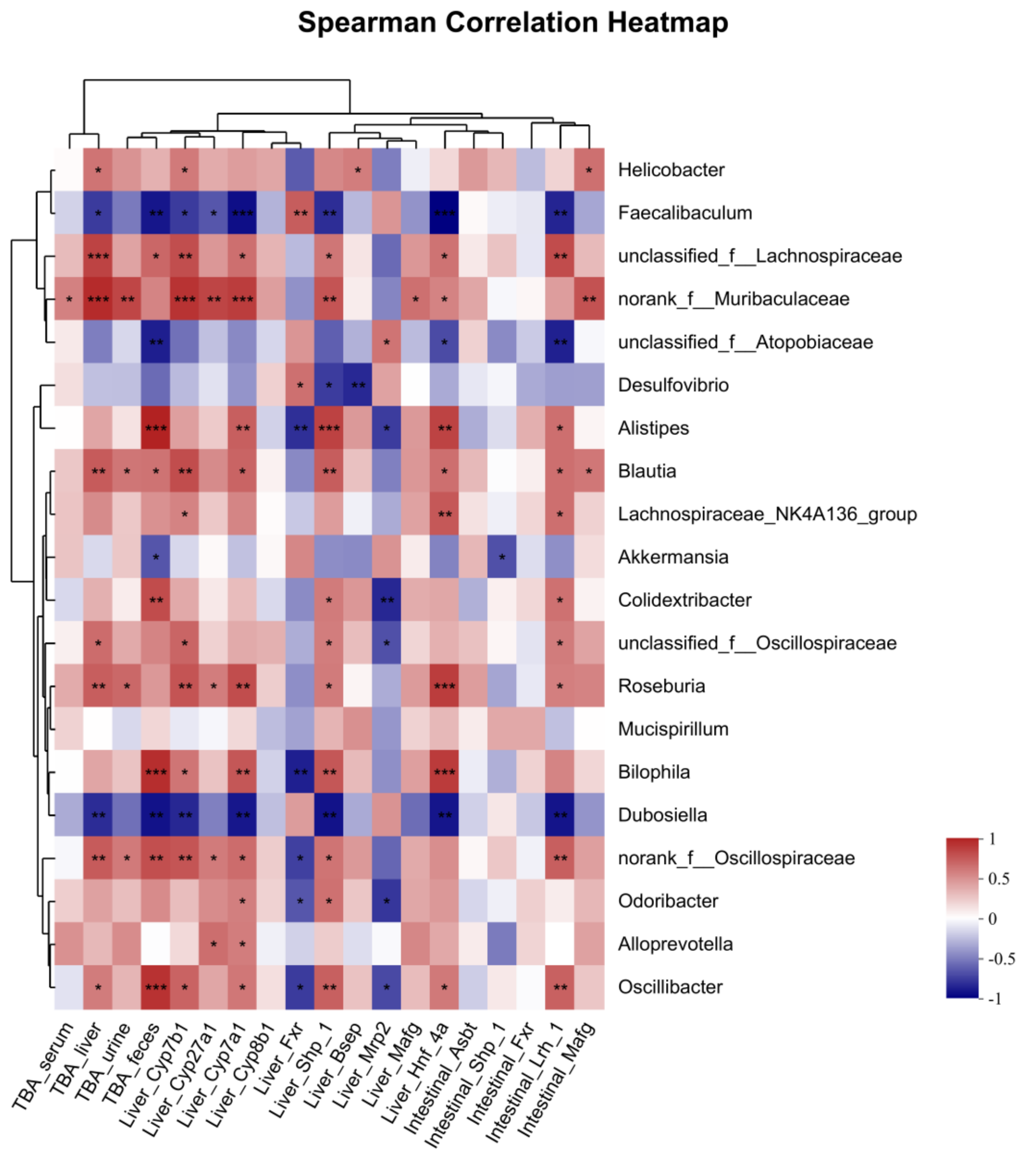

Figure S1. Correlation analysis between identified bacterial species with bile acid indexes and mRNA expression levels of bile acid metabolism-related genes. The color gradient is shown from blue (low abundance) to red (high abundance), and “*” indicates $p < 0.05$, “**” indicates $p < 0.01$, “***” indicates $p < 0.001$.

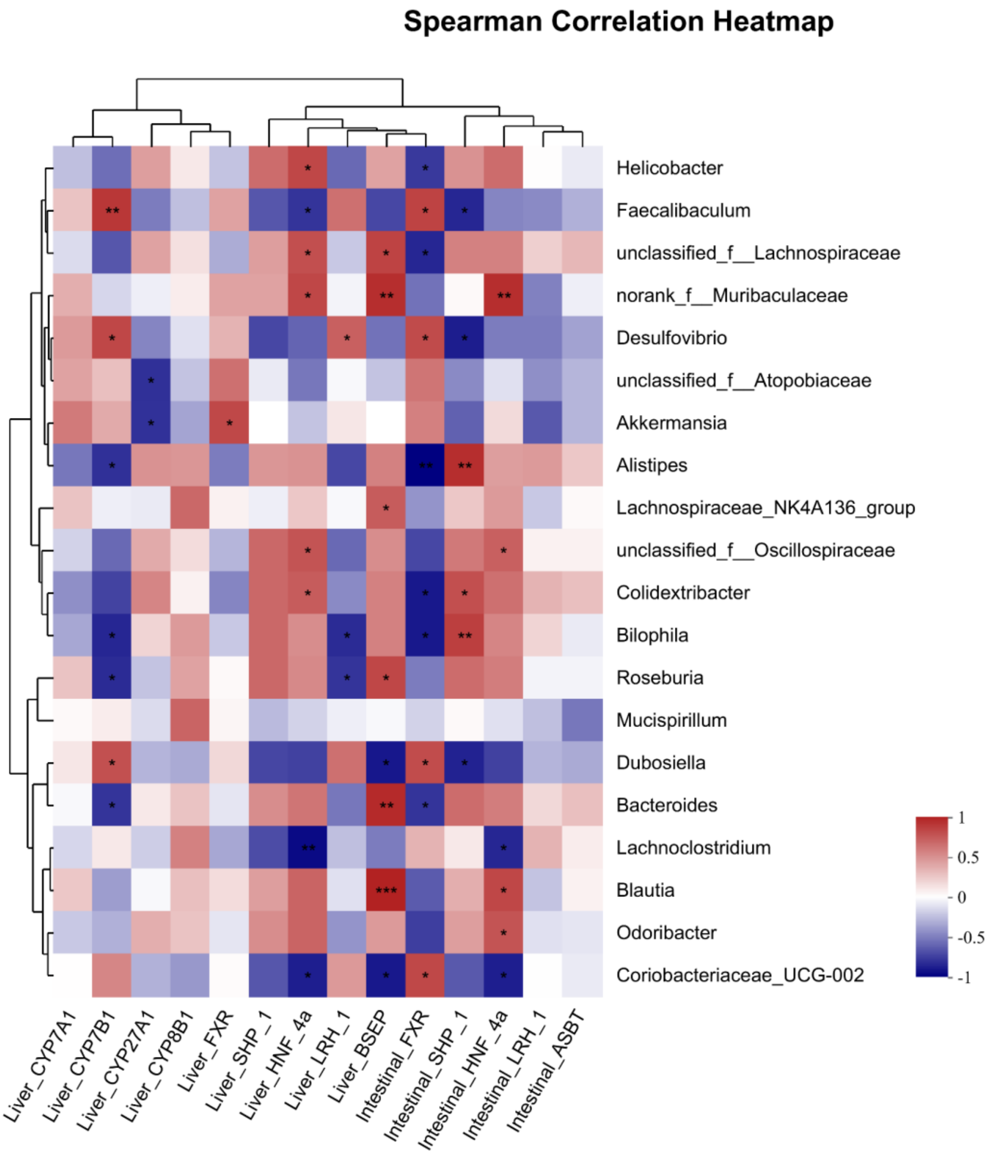

Figure S2. Correlation analysis between identified bacterial species with protein expression levels of bile acid metabolism-related genes. The color gradient is shown from blue (low abundance) to red (high abundance), and "*" indicates $p < 0.05$, "**" indicates $p < 0.01$, "***" indicates $p < 0.001$.

Table S1. Primer sequences used for real-time quantitative polymerase chain reaction (RT-qPCR).

Gene	Forward primer sequences	Reverse primer sequences
Abca1	AGTTTGACGCCATCACAGAGC	GCCCATTCACCAACCTTGC
Abcg1	GCTTGTTGGCCTCAGTTAAGG	GTAGCTCAGGCGTACAGAGAT
Abcg5	TCAATGAGTTTTACGGCCTGAA	GCACATCGGGTGATTTAGCA
Abcg8	TGCCCACCTTCCACATGTC	ATGAAGCCGGCAGTAAGGTAGA
Asbt	GTCTGTCCCCCAAATGCAACT	CACCCCATAGAAAACATCACCA
Bsep	GGACAATGATGTGCTTGTGG	CACACAAAGCCCCTACCAGT
Cyp7a1	AGCAACTAAACAACCTGCCATG CCAGTACT	GTCCGGATATTCAAGGATGCA
Cyp7b1	CCCTGCGTGACGAAATTGAC	AGAATAGTGCTTTCCAGGCAGA
Cyp8b1	TTGCAAATGCTGCCTCAACC	AGTGGGAAATTAACAGTCGCA
Cyp27a1	GCCTTGGAAGCCATCACCTA	AGATCTGATGAAGGCGGCAG
Fgf15	ACGTCCTTGATGGCAATCG	GAGGACCAAAACGAACGAAATT
Fxr	TGAGAACCCACAGCATTTCG	GCGTGGTGATGGTTGAATGTC
Hmgcr	GGCATTTGACAGCACTAGCA	CTTTGCATGCTCCTTGAACA
Hnf4α	CACGCGGAGGTCAAGCTAC	CCCAGAGATGGGAGAGGTGAT
Ldlr	GACACCAAGGGCGTAA	TGGAATCAACCCAATAGA
Lrh1	TGAGGAACAACTCCGGGAAAA	CAGACACTTTATCGCCACACA
Lxr	CTCAATGCCTGATGTTTCTCCT	TCCAACCCTATCCCTAAAGCAA

Mafg	ATGACGACCCCCAATAAAGGA	CACCGACATGGTTACCAGC
Mrp2	GTGTGGATTCCCTTGGGCTTT	CACAACGAACACCTGCTTGG
Npc1l1	TTTCTAGGGGCCCTGACCTC	TTGAAAAGCAGCACACGACG
Shp1	CGATCCTCTTCAACCCAGATG	AGGGCTCCAAGACTTCACACA
Srbi	TGTACTGCCTAACATCTTGGTCC	ACTGTGCGGTTCATAAAAGCA
Srebp2	CAAGAAGAAGGCTGGAGAC	CACCACCGACAGATGATG
β-actin	ACCCCAGCCATGTACGTAGC	GTGTGGGTGACCCCGTCTC

Abca1: ATP-binding cassette transporter A1; *Abcg1*: ATP-binding cassette transporter G1; *Abcg5*: ATP-binding cassette subfamily G member 5; *Abcg8*: ATP-binding cassette subfamily G member 8; *Asbt*: apical sodium–dependent bile acid transporter; *Bsep*: bile salt export pump; *Cyp7a1*: cholesterol 7α-hydroxylase; *Cyp7b1*: oxysterol 7α-hydroxylase; *Cyp8b1*: cytochrome P450 8b1; *Cyp27a1*: cytochrome P450 27a1; *Fgf15*: fibroblast growth factor 15; *Fxr*: farnesoid X receptor; *Hmgcr*: 3-hydroxy-3-methyl glutaryl coenzyme A reductase; *Hnf4α*: hepatocyte nuclear factor 4α; *Ldlr*: low density lipoprotein receptor; *Lrh1*: liver receptor homolog-1; *Lxr*: liver X receptor; *Mafg*: MAF BZIP transcription factor G; *Mrp2*: multidrug resistance-associated protein 2; *Npc1l1*: niemann-pick C1-like 1; *Shp1*: small heterodimer partner-1; *Srbi*: scavenger receptor class B type I; *Srebp2*: sterol-regulatory element binding protein 2.

Table S2. Effects of neoagarotetraose (NAT) on body, liver, and fat weights in HFHCD-fed ApoE$^{-/-}$ mice.

	Body weight (g)	Liver weight (g)	Epididymal fat (g)	Perinephric fat (g)	Subcutaneous fat (g)
Normal	27.05±1.16 ^{c}	1.04±0.08 ^{c}	0.31±0.05 ^{b}	0.04±0.01 ^{b}	0.12±0.03 ^{c}
ApoE$^{-/-}$	31.20±1.05 ^{b}	1.29±0.10 ^{b}	0.38±0.09 ^{ab}	0.06±0.01 ^{a}	0.14±0.02 ^{b}
HFHCD	32.88±1.02 ^{a}	1.48±0.07 ^{a}	0.43±0.04 ^{a}	0.07±0.02 ^{a}	0.18±0.02 ^{a}
HFHCD+NAT	30.21±1.37 ^{b}	1.33±0.07 ^{b}	0.32±0.04 ^{b}	0.06±0.02 ^{ab}	0.12±0.04 ^{bc}

Data are presented as means ± SD (n = 10). Results marked with different letters are significantly different ($p < 0.05$).

Table S3. Impact of neoagarotetraose (NAT) supplementation on the gut microbiota composition at phylum levels.

	Relative abundance (%)						
	Firmicutes	Bacteroidota	Campilobacterota	Actinobacteriota	Desulfobacterota	Verrucomicrobiota	Deferribacterota
ApoE$^{-/-}$	46.22±13.25	26.81±11.59 ^{a}	19.01±10.68 ^{ab}	1.59±1.64 ^{b}	3.95±1.23	0.37±0.62	1.55±0.96
HFHCD	52.22±14.11	4.63±3.47 ^{b}	9.92±10.69 ^{b}	16.81±8.09 ^{a}	7.60±6.85	6.58±10.91	1.45±2.11
HFHCD+NAT	42.63±12.21	21.58±2.42 ^{a}	22.74±8.69 ^{a}	4.25±3.10 ^{b}	4.69±1.53	1.90±1.89	1.57±1.29

Data are presented as means ± SD (n = 5). Results marked with different letters are significantly different ($p < 0.05$).

Table S4. Impact of neoagarotetraose (NAT) supplementation on the gut microbiota composition at genus levels.

	Relative abundance (%)							
	Helicobacter	*Faecalibaculum*	*Unclassified_f_ Lachnospiraceae*	*norank_f_ Muribaculaceae*	*unclassified_f_ Atopobiaceae*	*Desulfovibrio*	*Alistipes*	*Blautia*
$ApoE^{-/-}$	19.01±10.68	1.58±1.88 [b]	14.77±6.04 [a]	11.21±4.74 [a]	1.37±1.65 [b]	1.17±0.38 [b]	7.62±6.16 [a]	5.11±1.93 [a]
HFHCD	9.91±3.70	34.10±12.06 [a]	2.64±2.01 [b]	2.42±2.08 [b]	11.82±7.57 [a]	7.54±2.64 [a]	0.64±0.37 [b]	0.26±0.29 [b]
HFHCD+NAT	22.74±8.69	1.39±0.96 [b]	15.88±5.76 [a]	14.37±2.78 [a]	3.83±3.04 [ab]	2.52±1.45 [b]	1.98±0.48 [ab]	3.96±1.63 [a]

Data are presented as means ± SD (n = 5). Results marked with different letters are significantly different ($p < 0.05$).
